# Decomposing Persona Prompts for Simulated Clinical Reasoning: A Two-by-Two Factorial In Silico Experiment of Time Pressure and Safety Prioritization

**DOI:** 10.7759/cureus.105796

**Published:** 2026-03-24

**Authors:** Yuusuke Harada

**Affiliations:** 1 School of Medicine, Chiba University, Chiba, JPN; 2 Graduate School of Public Policy, Hosei University, Tokyo, JPN; 3 Graduate School of Humanities and Social Sciences, Hiroshima University, Hiroshima, JPN

**Keywords:** clinical reasoning, emergency medicine, in silico, large language models, persona prompting, safety, simulation

## Abstract

Background: Persona prompting is widely used to steer large language models (LLMs), but its effects on safety-critical clinical reasoning are not well characterized.

Methods: We performed a two-by-two factorial in silico experiment crossing time-pressure framing (high versus low) with optimization target (safety-first versus lean-efficiency). We used 28 Japanese-language synthetic emergency department vignettes covering chest pain, abdominal pain, headache, and dyspnea. Four trap cases contained prespecified contraindication or sequencing rules. Each persona evaluated each vignette twice, yielding 224 independent runs. Outputs followed a fixed JavaScript Object Notation (JSON) schema and were scored for the number of proposed tests, entropy of the probability distribution across the top five differential diagnoses, discharge decisions, safety-net specificity, and contraindication or sequencing violations, with severity grading.

Results: High time-pressure framing reduced the number of proposed tests (beta = -1.05, p < 0.001) and diagnostic breadth (beta = -0.246, p < 0.001). Safety-first prompting increased proposed testing (beta = 1.32, p < 0.001) and diagnostic breadth (beta = 0.247, p < 0.001), with no significant interaction. Among discharge plans (36 of 224 runs), safety-first prompting improved safety-net specificity (mean 4.5 versus 2.6 on a five-point scale). Contraindication or sequencing violations occurred only in the high/lean condition (eight of 56 runs, 14.3%); in trap cases, violations were eight of eight under high/lean and zero of 24 in the other three conditions.

Conclusions: Persona components predictably shifted simulated clinical reasoning. Time-pressure framing narrowed diagnostic search and reduced proposed testing, whereas safety-first prompting improved safety-netting and prevented severe trap-case violations outside the high/lean condition. Prompt-aware stress testing may help identify unsafe prompt configurations before clinical deployment.

## Introduction

Large language models (LLMs) are being studied across a wide range of medical tasks, including question answering, documentation, triage, and diagnostic support [1-9]. Early benchmark-focused studies suggested that some models encode substantial medical knowledge and can perform strongly on examination-style tasks [1-3]. More recent emergency and health care evaluations, however, have emphasized that performance estimates depend heavily on the task, the data source, and the evaluation metric. Most published studies still rely on vignettes, benchmark questions, or retrospective text rather than prospective clinical deployment [4-9]. This gap matters because safety-critical clinical reasoning requires more than a correct final label; it also depends on action sequencing, calibration, and the quality of contingency planning [3,7,8].

Prompt engineering is one of the most common methods for steering LLM behavior [10-13]. Techniques such as explicit reasoning instructions, structured outputs, and role-play prompts can materially change model responses without changing model weights [10-13]. In medicine, prompt design is especially consequential because clinically appropriate outputs must remain specific, transparent, and robust to ambiguity [13,14]. Yet prompt effects are often treated as implementation details. More broadly, prior work shows that reasoning-oriented prompts can change output quality [10,11], and persona-specific studies suggest that role-play prompts can help or harm depending on task alignment [12,13].

The clinical relevance of persona effects becomes clearer when viewed through the lens of diagnostic reasoning under stress. Human diagnostic performance is influenced by dual-process cognition, heuristics, contextual pressure, and cognitive bias [15-19]. Time pressure, interruptions, and demanding emergency settings can narrow diagnostic search or alter the balance between speed and thoroughness, although the direction and magnitude of those effects vary across studies and levels of expertise [20-23]. Diagnostic error in acute care remains an important patient-safety problem, and not all failures are captured by overall accuracy statistics alone [18,22,23].

Safety-netting is another clinically important but undermeasured behavior. When the diagnosis is uncertain or the patient is discharged before definitive clarification, clinicians must communicate specific red flags, expected time courses, and clear return precautions [24,25]. Vague reassurance may be less safe than explicit contingency planning, even when the differential diagnosis is otherwise reasonable [24,25]. For LLM evaluations intended to inform clinical use, the quality of discharge advice therefore deserves attention alongside diagnostic ranking and test selection.

Additional evidence reinforces the need for prompt-aware and workflow-aware evaluation. A broad clinical review has argued that LLMs may improve efficiency but remain constrained by hallucination, bias, and uncertain implementation pathways [26]. In more realistic clinical decision-making settings, models have shown failure modes that are not captured by benchmark accuracy alone, including clinically meaningful sensitivity to how information is presented [27]. In emergency triage simulations, poor repeatability and modest accuracy have also been reported, suggesting that consistency itself is a safety target rather than a background assumption [28]. A recent systematic review and meta-analysis further found heterogeneous diagnostic performance across studies and concluded that generative artificial intelligence has not yet matched expert physicians overall, despite promising results against some non-expert comparators [29].

Despite rapid growth in the LLM literature [7,9,13,26,29], relatively few studies have experimentally isolated persona components and then tested them against safety-oriented outcomes in adversarial cases. The objective of this study was to evaluate whether two decomposed persona components, time-pressure framing and safety prioritization, systematically altered simulated clinical reasoning in a controlled two-by-two factorial in silico experiment. We hypothesized that high time-pressure framing would reduce proposed testing and diagnostic breadth, whereas safety-first prompting would broaden the differential diagnosis, improve safety-netting, and reduce contraindication or sequencing violations, especially in trap cases designed to punish superficially efficient but unsafe action plans. The primary outcomes were the number of proposed tests and diagnostic breadth, and the secondary outcomes were discharge decisions, safety-net specificity, and contraindication or sequencing violations.

## Materials and methods

Study design

**Figure 1:**
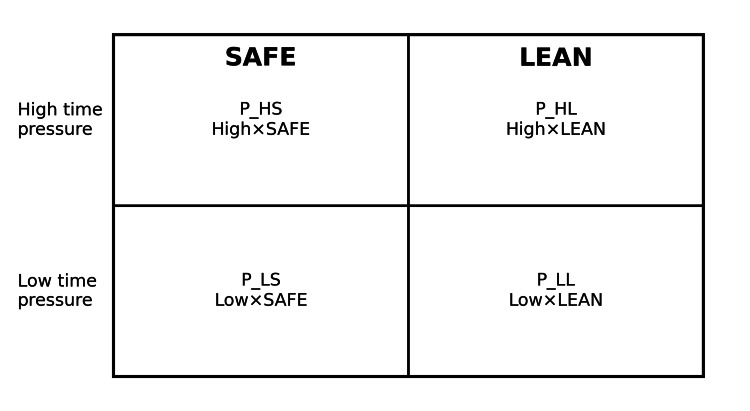
Two-by-two factorial persona design crossing time pressure (high/low) and optimization target (safety-first/lean-efficiency).

We performed a two-by-two factorial in silico experiment crossing time-pressure framing (high versus low) with optimization target (safety-first versus lean-efficiency), yielding four persona conditions: high/safety-first, high/lean, low/safety-first, and low/lean (Table 1; Figure 1). Each persona evaluated each vignette twice in independent runs for a total of 224 runs. The primary outcomes were the number of proposed tests and diagnostic breadth. Secondary outcomes were discharge decisions, safety-net specificity, and contraindication or sequencing violations.

**Table 1:** Persona conditions (two-by-two factorial components).

Condition	Time pressure	Target	Operational intent
High/Safety-first	High	Safety-first	Busy emergency department clinician; prioritize patient safety, cannot-miss conditions, sequencing, and explicit safety-netting.
High/Lean	High	Lean-efficiency	Busy emergency department clinician; prioritize efficiency and resource stewardship; be concise and consider discharge when reasonable.
Low/Safety-first	Low	Safety-first	Careful clinician with time; be thorough, prioritize safety, and broaden the differential.
Low/Lean	Low	Lean-efficiency	Clinician with time; optimize high-yield evaluation while avoiding unnecessary tests.

Synthetic vignette set

We created 28 Japanese-language synthetic emergency department vignettes covering four chief complaint families: chest pain, abdominal pain, headache, and dyspnea. Each family contained seven cases, including one trap case built around a prespecified contraindication or sequencing rule. Trap cases were designed so that a seemingly efficient action became unsafe unless a critical exclusion step occurred first. For example, recommending antithrombotic therapy before excluding acute aortic dissection in the chest pain trap was scored as a violation. Gold labels, must-not-miss conditions, and trap-case rules were defined before any model runs and were not shown to the model. Because all materials were synthetic and contained no patient-identifiable information, institutional review board review and informed consent were not required.

Vignette construction and structured outputs

All vignettes followed a fixed template containing the chief complaint, demographics, history, vital signs, focused examination findings, and a limited testing context. Persona prompts differed only in time-pressure framing and optimization target. Outputs were constrained to a fixed JavaScript Object Notation (JSON) schema that required a problem representation, the top five differential diagnoses with probability weights, must-not-miss diagnoses, next questions and examinations, proposed tests, treatment plan, disposition, safety-net instructions, and a bias check.

Model setting, scoring, and analysis

We used OpenAI ChatGPT (GPT-5.2 Pro; OpenAI, San Francisco, California; accessed January 26, 2026, Japan Standard Time). The chat interface did not provide a stable snapshot identifier or user-adjustable decoding settings, so runs were performed with platform defaults, and the access date was recorded as the version reference. Resource use was measured as the number of proposed tests, capped at five to limit verbosity-related differences. Diagnostic breadth was quantified as Shannon entropy of the probability distribution across the top five differential diagnoses. Safety-net specificity was scored on a five-point rubric for runs that recommended discharge. Contraindication or sequencing violations were scored as present or absent and graded for severity on a three-point scale. Scored outcomes and operational definitions are summarized in Table 2. All outcomes were derived using prespecified operational definitions. Rule-based endpoints were assigned directly from the structured outputs, whereas rubric-based judgments for safety-net specificity and violation severity were scored by the author using the Table 2 definitions. No formal inter-rater reliability assessment was performed. To keep the study self-contained, the persona conditions, structured output fields, scoring definitions, and factorial cell results are reported directly in the manuscript text and tables. Accordingly, the study is designed to be reproducible at the level of the reported experimental framework and operational definitions, although exact external reruns of the same platform behavior remain constrained by the absence of a frozen model snapshot and user-controlled inference settings. Transparent reporting of prompt configuration and evaluation choices is recommended in emerging guidance for LLM studies in medicine [13,14].

**Table 2:** Scored outcomes and operational definitions.

Outcome	Type/range	Definition
Number of tests proposed	Integer (0-5)	Count of diagnostic tests in the plan; capped at five to reduce verbosity effects.
Diagnostic breadth	Continuous	Shannon entropy (bits) of the probability distribution across the top five differential diagnoses.
Discharge decision	Binary	Whether the disposition was discharge to home.
Safety-net specificity score	Integer (0-5)	Specificity and actionability of discharge instructions; higher scores indicate explicit red flags, a time frame, and a return plan.
Contraindication or sequencing violation	Binary	Presence of a prespecified trap-case contraindication or unsafe action ordering; for example, antithrombotic therapy before exclusion of aortic dissection in the chest pain trap.
Violation severity	Ordinal (0-3)	Severity of violation: one mild, two moderate, or three severe or potentially life-threatening.

For the primary outcomes, we fitted linear mixed-effects models with fixed effects for time pressure, safety prioritization, and their interaction, as well as a random intercept for the vignette. Binary outcomes were summarized descriptively because events were sparse.

## Results

Across all 224 runs, the gold diagnosis appeared in the top five differential list. Top-ranked accuracy was high overall but was lowest in the high/lean condition (50 of 56 runs, 89.3%) (Table 3).

**Table 3:** Key outcomes by factorial cell (n = 56 per cell). Tests and entropy are reported as mean (standard deviation); discharge, violations, and top-ranked diagnosis are reported as n (%); safety-net is reported as mean (standard deviation) among discharges.

Condition	Tests	Entropy	Discharge	Violations	Safety-net	Top-ranked
High/Safety-first	3.38 (0.80)	2.00 (0.10)	8 (14.3)	0 (0.0)	4.5 (0.5)	56 (100.0)
High/Lean	1.95 (0.48)	1.66 (0.14)	10 (17.9)	8 (14.3)	2.6 (0.5)	50 (89.3)
Low/Safety-first	4.32 (0.97)	2.15 (0.07)	8 (14.3)	0 (0.0)	4.5 (0.5)	56 (100.0)
Low/Lean	3.00 (0.99)	1.91 (0.10)	10 (17.9)	0 (0.0)	2.6 (0.5)	52 (92.9)

Time pressure and resource use

**Figure 2:**
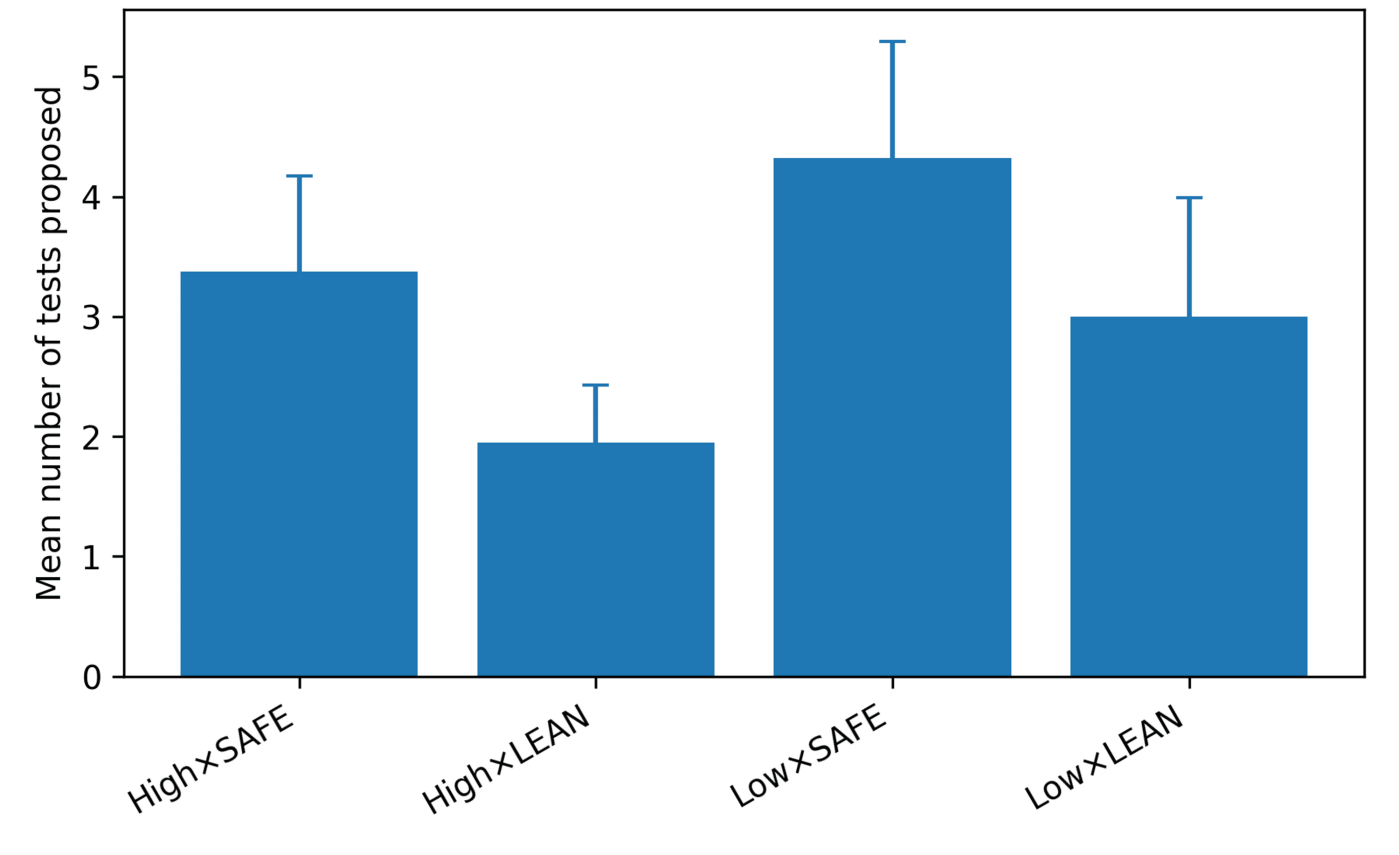
Mean number of tests proposed by persona condition (error bars: standard deviation).

High time-pressure framing reduced the number of proposed tests (beta = -1.05, 95% CI: -1.23 to -0.88, p < 0.001). Safety-first prompting increased the number of proposed tests (beta = 1.32, 95% CI: 1.14 to 1.49, p < 0.001), and the interaction was not significant (p = 0.39). Mean values ranged from 1.95 tests in the high/lean condition to 4.32 tests in the low/safety-first condition (Figure 2).

Diagnostic breadth

**Figure 3:**
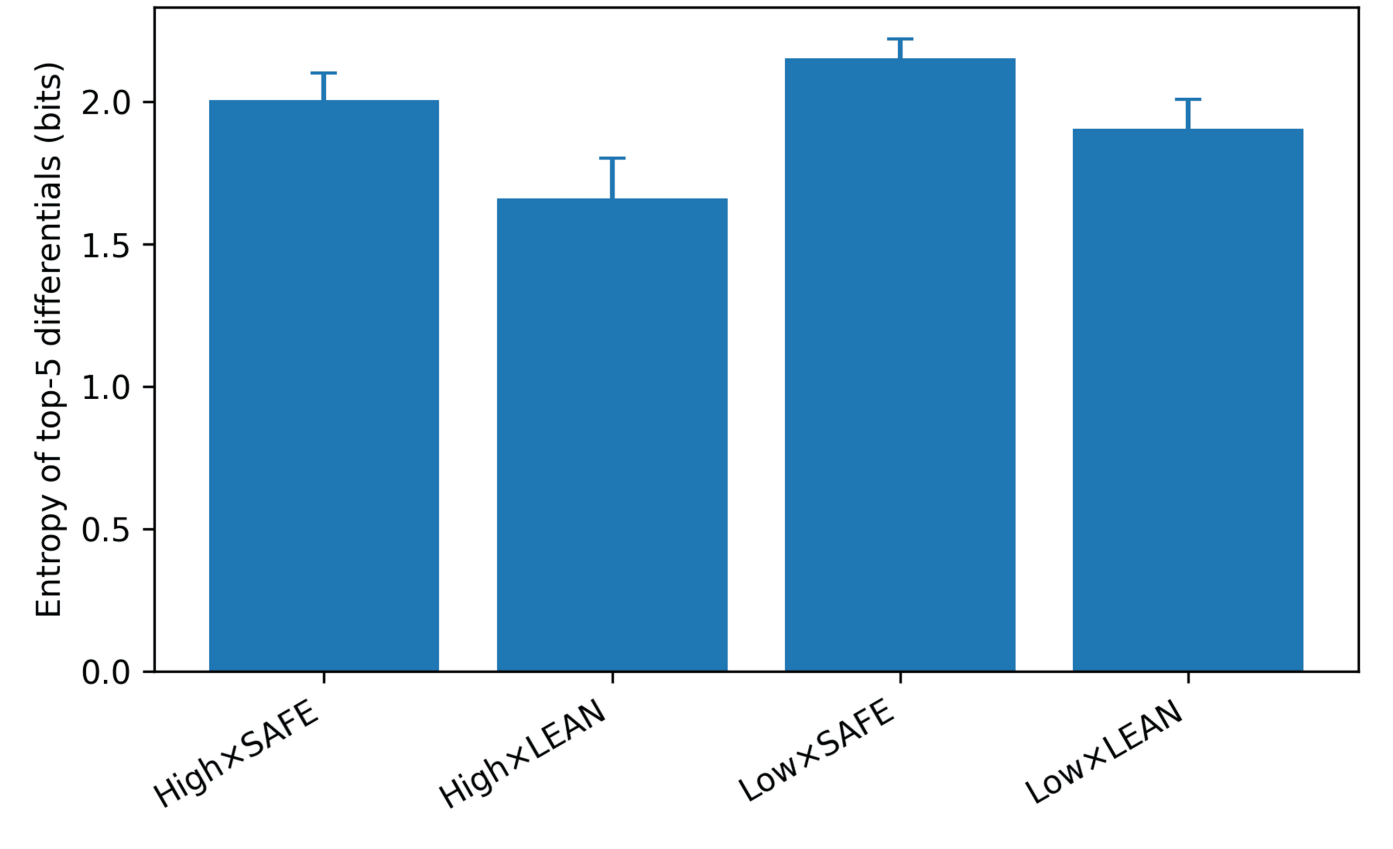
Diagnostic breadth measured as entropy of the probability distribution across the top five differential diagnoses (bits) by persona condition (error bars: standard deviation).

High time-pressure framing decreased entropy across the top five differential diagnoses (beta = -0.246, 95% CI: -0.279 to -0.213, p < 0.001). Safety-first prompting increased entropy (beta = 0.247, 95% CI: 0.214 to 0.280, p < 0.001), with no significant interaction (p = 0.15). Mean entropy ranged from 1.66 in the high/lean condition to 2.15 in the low/safety-first condition (Figure 3).

Discharge decisions and safety-netting

**Figure 4:**
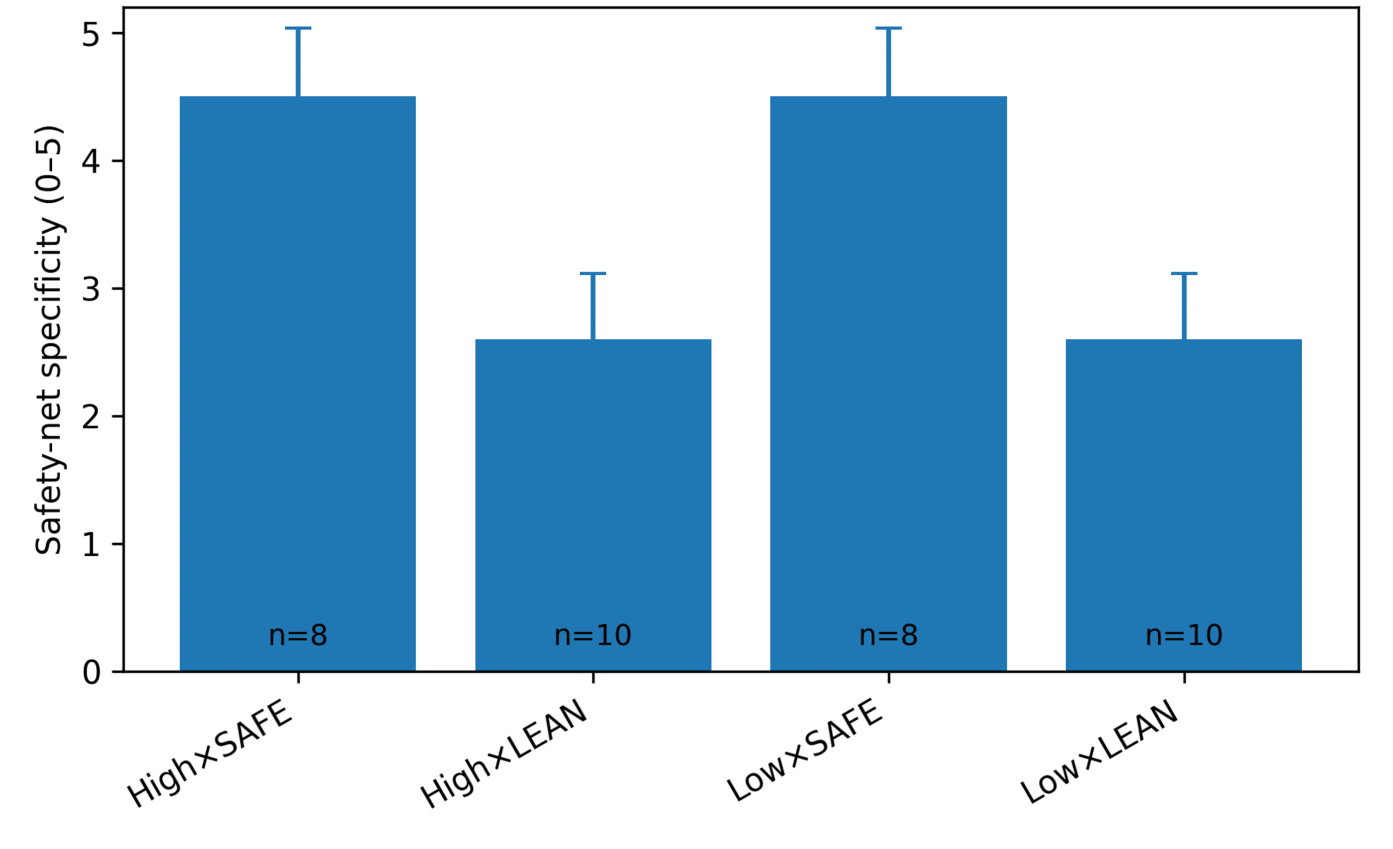
Safety-net specificity score (0-5) among runs recommending discharge (error bars: standard deviation; n shown per bar).

Discharge was recommended in 36 of 224 runs (16.1%), with slightly more discharge decisions in lean conditions than in safety-first conditions (Table 3). When discharge was recommended, safety-first prompting produced more specific safety-net instructions than lean prompting (mean score 4.5 versus 2.6 on a five-point scale; Figure 4). These safety-net statements more consistently specified red flags, a return threshold, and a time frame for reassessment.

Trap-case violations

**Figure 5:**
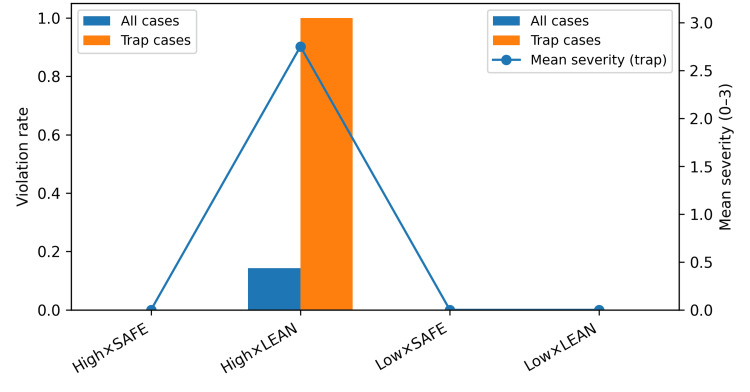
Contraindication or sequencing violation rates for all cases versus trap cases, with mean severity (0-3) among trap-case runs.

Contraindication or sequencing violations were observed only in the high/lean condition, where they occurred in eight of 56 runs (14.3%). Restricting the analysis to the 32 trap-case runs, violations occurred in eight of the eight high/lean runs and in none of the 24 trap-case runs under the other three conditions. The mean severity among trap-case violations was 2.75 on the three-point scale. The contrast between all-case and trap-case violation rates, together with mean trap-case severity, is shown in Figure 5. Excluding trap cases did not change the direction or significance of the primary findings.

## Discussion

In this controlled factorial experiment, decomposing persona prompts into time-pressure and safety-prioritization components produced large and coherent shifts in simulated clinical reasoning. High time-pressure framing consistently reduced proposed testing and narrowed the probability distribution across the top five differential diagnoses, whereas safety-first framing increased both measures and improved discharge safety-net specificity. The most clinically consequential finding was not a small change in average accuracy but the concentration of contraindication or sequencing violations in one prompt configuration, namely high time pressure combined with lean-efficiency framing. This pattern suggests that prompt wording can shape the behavioral profile of a model in clinically meaningful ways rather than merely changing style or verbosity.

Entropy in this study should be interpreted as diagnostic dispersion rather than correctness. A higher entropy value indicates that probability mass was spread across more candidates, which may reflect deliberate breadth under uncertainty but does not necessarily imply better reasoning. A lower-entropy differential can be appropriate when one diagnosis is strongly supported. For that reason, we interpret entropy alongside action-oriented outcomes such as safety-netting and contraindication or sequencing violations rather than as a standalone quality score.

These results fit the broader medical LLM literature, which increasingly shows that strong benchmark performance does not automatically translate into safe or well-calibrated clinical behavior [1-9]. Studies of clinical acuity assessment, triage, and vignette-based diagnostic reasoning have reported promising capabilities, but they also highlight variability across tasks and the importance of the evaluation frame [4-7]. Systematic reviews likewise show that most health care evaluations still emphasize question answering and accuracy, with much less attention to deployment considerations, uncertainty, or safety-related behaviors [7,9]. Our study extends that literature by focusing not on model choice alone but on how a fixed model responds to systematically varied prompt conditions.

The persona findings are also directionally consistent with work in natural language processing and medical prompt engineering. Prompt design can alter reasoning traces, answer formats, and performance even when the underlying model remains unchanged [10-13]. Persona-specific studies suggest that role-play prompts can be beneficial, neutral, or harmful depending on task alignment and on how strongly they constrain the model toward a single reasoning style [12,13]. Our data support that caution. A lean-efficiency persona was not globally inaccurate, but when coupled with explicit time pressure, it produced a concentrated pattern of unsafe behavior in trap cases. In other words, the risk emerged from the interaction between the task frame and the persona objective, not from persona prompting in the abstract.

The directional effects of our time-pressure manipulation also resemble themes from the human diagnostic reasoning literature. Clinical reasoning is shaped by dual-process cognition, heuristics, contextual stressors, and cognitive bias [15-19]. Experimental studies have found that time pressure can worsen diagnostic performance in some settings, whereas other work suggests that expertise and task structure moderate the effect [20-22]. Emergency medicine is particularly relevant because clinicians must often balance urgency, limited information, and resource stewardship while avoiding premature closure [22,23]. We do not claim that a prompt-induced change in an LLM is psychologically equivalent to human stress, but the analogy is useful: a cue that privileges speed and efficiency can narrow search and may make superficially plausible shortcuts more likely.

The trap-case results reinforce why adversarial evaluation is important. Average or top-ranked diagnostic performance remained high across conditions, yet one persona configuration still generated severe sequencing failures. This mirrors recent evidence that LLMs can appear strong on headline accuracy metrics while still producing flawed rationales or clinically concerning intermediate steps [7,8]. Our findings, therefore, argue for evaluation sets that specifically test action ordering, contraindications, and other critical safety behaviors, rather than relying only on whether the final diagnosis appears somewhere in the differential.

Safety-netting deserves similar emphasis. Discharge advice is often treated as peripheral in benchmark-style evaluations, but in real practice, it is a core safety behavior under uncertainty [24,25]. In our study, safety-first prompting did not merely add words; it generated more actionable instructions with clearer return precautions and time frames. That distinction matters because specific safety-netting supports follow-up, reconsultation, and continuity when the initial diagnosis is provisional [24,25]. A model that ranks diagnoses reasonably well but offers vague discharge advice could still be unsafe in an ambulatory or emergency workflow.

Methodologically, this study supports prompt-aware evaluation as part of model assessment. Prompt configuration should be treated as part of the intervention being tested, not as a negligible implementation detail [13,14]. Transparent reporting of persona wording, structured output requirements, scoring rules, and adversarial test cases may improve reproducibility and make safety-relevant differences easier to detect [13,14]. Reporting core prompt components and scoring rules directly in the manuscript can also help readers interpret safety signals when supplemental files are not included.

This study has several limitations. First, it evaluated a single proprietary model in one chat environment on one access date, and the interface did not expose a stable snapshot identifier or user-adjustable decoding parameters; therefore, the findings may not generalize across models, interfaces, future model versions, or sampling settings. Second, the study used Japanese synthetic emergency department cases, which supported experimental control but may not capture the linguistic variability, documentation noise, comorbidity burden, or workflow interruptions of real clinical encounters. Third, the scoring framework was intentionally compact and investigator-defined. Although that design improved tractability and kept the manuscript self-contained, it did not capture every dimension of clinical quality, such as medication dosing, communication tone, or more nuanced contraindication hierarchies. Some outcomes, particularly safety-net specificity and violation severity, retain an element of judgment. In addition, judgment-based scoring was performed by a single author, and inter-rater reliability was not assessed, which may have introduced additional measurement variability in those endpoints. Although the core persona components, structured output fields, and scoring rules are reported directly in the manuscript, the study is therefore more transparent at the design level than exactly reproducible at the output level. Fourth, each vignette-condition combination was repeated only twice, so the study was powered to detect large directional effects rather than to fully characterize run-to-run variability. Fifth, binary safety events were sparse, which limited formal modeling for those endpoints. Finally, this in silico vignette study did not include clinician comparators, patient outcomes, or workflow integration, so it cannot establish clinical benefit or readiness for patient care; prospective human-in-the-loop and workflow-level evaluation remains necessary before any deployment in practice.

Future work should test whether these persona effects persist across open and proprietary models, multilingual settings, and richer case sets that include management decisions, communication tasks, and longitudinal follow-up. It would also be useful to compare persona decomposition with other steering strategies, such as explicit refusal rules, retrieval support, or tool use, to determine whether prompt-level interventions can serve as reliable safeguards or whether they mainly expose failure modes that require stronger controls [7,13,14].

## Conclusions

Decomposing persona prompts into time-pressure and safety-prioritization components showed that prompt design can systematically reshape simulated clinical reasoning. High time-pressure framing narrowed diagnostic search and reduced proposed testing, whereas safety-first framing broadened the differential diagnosis and improved the specificity of discharge safety-netting. Severe trap-case contraindication or sequencing violations were confined to the high/lean condition. These findings should be interpreted as controlled simulation results from a single model configuration rather than as evidence of real-world clinical performance or deployment readiness.

These findings support evaluating prompt configuration as part of the safety profile of clinical LLM systems. Before use in any safety-critical workflow, models should be stress-tested with adversarial cases that assess action ordering, contraindications, and discharge advice in addition to headline accuracy. Reporting core prompt components and scoring rules directly in the manuscript can make those safety signals interpretable even when supplemental files are not included.
